# Serum Cystatin C within 24 hours after admission: a potential predictor for acute kidney injury in Chinese patients with community acquired pneumonia

**DOI:** 10.1080/0886022X.2023.2194444

**Published:** 2023-03-28

**Authors:** Dawei Chen, Linglin Jiang, Yan Tan, Jing Zhao, Wenjuan Huang, Binbin Pan, Xin Wan

**Affiliations:** a Department of Nephrology, Nanjing First Hospital, Nanjing Medical University, Nanjing, P.R. China; b Department of Respiratory Medicine, Nanjing First Hospital, Nanjing Medical University, Nanjing, P.R. China

**Keywords:** Cystatin C, biomarker, acute kidney injury, community-acquired pneumonia

## Abstract

**Background:**

Acute kidney injury (AKI) is common in patients with community-acquired pneumonia (CAP), and is associated with poor prognosis. Therefore, in this study, we evaluated whether AKI in Chinese patients with CAP could be well predicted by serum Cystatin C within 24 h after admission.

**Methods:**

Univariate and multivariate logistic regression analyses were used to investigate independent factors of AKI in patients with CAP.

**Results:**

Totally, 2716 patients with CAP were included in this study. 766 (28%) patients developed AKI. After multivariate logistic regression analysis, serum Cystatin C (odds ratio [OR] 4.27, 95% confidence interval [CI] 3.36–5.44; *p* < 0.001) was an independent factor for AKI in patients with CAP. Serum Cystatin C had an area under the receiver operating characteristic curve (AUC) of 0.81 for predicting AKI, with an optimal cutoff value of 1.37 mg/L, computing 68% sensitivity, 80% specificity. Furthermore, serum Cystatin C within 24 h after admission still had a good and stable prediction efficiency for AKI in various subgroups (age, gender, hypertension, diabetes, coronary artery disease, cardiac insufficiency, cerebrovascular disease, atrial fibrillation, chronic obstructive pulmonary disease, chronic kidney disease, and tumor, albumin, anemia, platelet count, white blood cell count, and uric acid, confusion, uremia, respiratory rate, blood pressure, and age 65 years or older [CURB-65] score, acute respiratory failure, intensive care unit admission, and mechanical ventilation) of patients with CAP (AUCs: 0.69–0.84).

**Conclusion:**

Serum Cystatin C within 24 h after admission appears to be a good biomarker for predicting AKI in Chinese patients with CAP.

## 1. Introduction

Community-acquired pneumonia (CAP) is responsible for substantial mortality, with a third of patients dying within 1 year after being discharged from the hospital for pneumonia [1]. Acute kidney injury (AKI) is a common complication of CAP, with incidence rates from 18 to 34% [2–4]. Moreover, AKI is associated with a poor prognosis in patients with CAP [2,3,5,6]. Lakhmir S. et al. found that patients who were admitted to the hospital for pneumonia and developed AKI had a poor effect on long-term prognosis. They were at high risk for death, dialysis, and permanent loss of renal function [6]. Even among patients diagnosed with non-severe pneumonia, AKI could increase higher long-term mortality risk [3]. Recently, our team also found that patients with CAP who developed AKI had worse short-term prognosis. They were more likely to require admission to an intensive care unit, mechanical ventilation, invasive mechanical ventilation, or noninvasive mechanical ventilation, had higher in-hospital mortality, and experienced a longer duration of hospital stay [5].

Serum creatinine (SCr) is affected by multiple factors, such as muscle mass, dehydration, and dietary protein intake. [7], and the increase in SCr during AKI is delayed, which leads to deferred diagnosis [8]. However, unlike SCr, the level of serum Cystatin C is not affected by age, sex, body muscle mass, and diet [9]. A previous study showed that Cystatin C was a good biomarker in the prediction of AKI in other clinical settings [9], as it was not influenced by age, gender, race, muscle mass, and protein intake [10]. However, the evidence regarding the prediction efficiency of Cystatin C for AKI in Chinese patients with CAP is scarce. Therefore, in this study, we evaluated whether AKI in Chinese patients with CAP could be well predicted by serum Cystatin C within 24 h after admission.

## 2. Subjects, materials and methods

### 2.1. Patient selection

**Figure 1.**
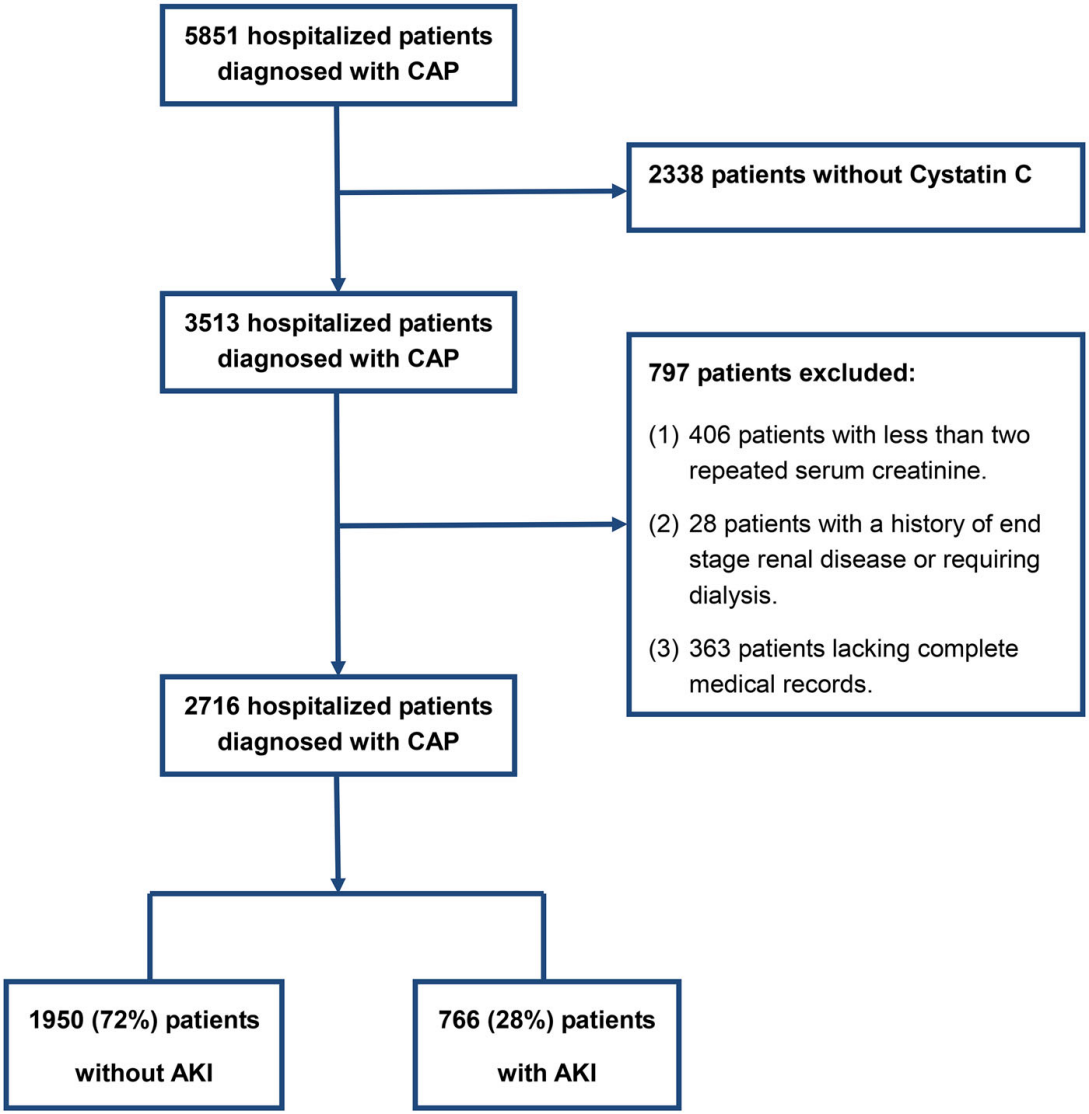
Flowchart for patient selection. CAP: community-acquired pneumonia; AKI: acute kidney injury.

This is a case-control study. We reviewed the medical records of 5851 patients, who were ≥18 years of age and admitted to the hospital for CAP at Nanjing First Hospital from January 2014 to May 2017. Exclusion criteria were as follows: patients without serum Cystatin C, patients with less than two repeated SCr, patients with a history of end-stage renal disease or requiring dialysis, and patients lacking complete medical records. Finally, 2716 patients were enrolled in this study. (Figure 1)

### 2.2. Definitions of CAP and AKI

Pneumonia is diagnosed based on the detection of interstitial infiltrate changes on chest radiography or CT in patients with one or more of (a) recent presence of dyspnea, cough, or sputum; (b) core body temperature > 38.0 °C; or (c) peripheral white blood cell > counts10 × 10^9^/L or < 4 × 10^9^/L. In addition, illness onset was specifically in the community, rather than in the healthcare setting [11,12].

The definition of AKI in our study adhered to the Kidney Disease Improving Global Outcomes (KDIGO) criteria, which defined AKI as an increase in SCr levels by ≥1.5-fold from baseline within 7 days of illness onset or an increase in SCr levels by ≥0.3 mg/dL (26.4 μmol/L) within 24 h of illness onset [13]. Baseline SCr values were defined as the lowest levels measured during hospitalization. Due to the lack of data concerning urine output, urine output standards were not considered in this study.

### 2.3. Data collection

Demographics (gender and age), comorbid conditions (hypertension, diabetes mellitus, coronary artery disease, cardiac insufficiency, atrial fibrillation, chronic obstructive pulmonary disease [COPD], chronic kidney disease, pulmonary hypertension, cerebrovascular diseases, and tumor), complication (acute respiratory failure [14]), severity scoring on admission (confusion, uremia, respiratory rate, blood pressure, and age 65 years or older [CURB-65]) [15], and laboratory tests (albumin, uric acid, serum Cystatin C, hemoglobin, platelet count, and white blood cell count) within 24 h after admission were collected from the hospital records.

### 2.4. Data analysis

Baseline characteristics were presented as means ± SDs or medians and interquartile ranges for continuous variables if appropriate, and proportions for categorical variables. Students’ *t* tests or Mann–Whitney tests were used to compare continuous variables between groups. Chi-square tests or Fisher exact were conducted to tell the differences in categorical variables between groups.

Univariate and multivariate logistic regression analysis were used to identify independent risk factors of AKI. The prediction performance of all independent factors for AKI was measured using the area under the receiver operating characteristic (ROC) curves (AUCs). We found that the maximum AUC was reported for serum Cystatin C within 24 h after admission. To determine the optimal cutoff value for serum Cystatin C in discriminating AKI, the Youden index was utilized to calculate the cutoff value (Youden index = sensitivity + specificity − 1, ranging from 0 to 1).

As a stratification analysis to evaluate the performance of serum Cystatin C within 24 h after admission for predicting AKI in various subgroups, we conducted a multivariate logistic regression analysis to explore whether serum Cystatin C was still an independent predictor for AKI in various subgroups. The various subgroups were classified by age, gender, comorbidities (hypertension, diabetes, coronary artery disease, cardiac insufficiency, cerebrovascular disease, atrial fibrillation, COPD, chronic kidney disease, and tumor), laboratory investigations (albumin, anemia [for male, hemoglobin <120 g/L; for female, hemoglobin <110 g/L] [16], platelet count, white blood cell count, and uric acid), CURB-65 Score, and complication (acute respiratory failure). Stratification analyses adjusted for all the above factors except the stratification factor itself. For the subgroups of intensive care unit (ICU) admission, and mechanical ventilation, stratification analyses adjusted for all the above factors (demographics, comorbid conditions, complications, and laboratory investigations). Furthermore, the prediction performance of serum Cystatin C for AKI was also measured by AUCs in various subgroups. P values < 0.05 were considered as statistically significant. Statistical analysis was using SPSS software version 22 (IBM, Armonk, NY, USA), the EmpowerStats (www.empowerstats.net, X&Y solutions, Inc. Boston MA) and R version 3.6.1 (http://www.r-project.org).

## 3. Results

### 3.1. Patient characteristics

Totally, 2716 patients were included in this study. The mean (median, range) age of the patients was 71.6 (75, 63–83) years and most of the study population consisted of males (60.9%). 487 (17.9%) patients complicated with acute respiratory failure. 447 (16.5%) patients required mechanical ventilation, and 597 (22.0%) patients needed admission to ICU.

### 3.2. AKI characteristics

766 (28%) patients developed AKI. The characteristics of patients with AKI were shown in Table 1. Compared with non-AKI group, male gender (67.2% versus 58.4%; *p* < 0.001) and older age (78.2 years versus 69.0 years; *p* < 0.001) had significant differences between the two groups. Hypertension, diabetes mellitus, coronary artery disease, cardiac insufficiency, atrial fibrillation, chronic kidney disease, and cerebrovascular diseases were more common in the AKI group. However, no statistically significant comorbidities were in COPD, pulmonary hypertension, and tumor between the two groups. Patients in the AKI group were more commonly complicated with acute respiratory failure (39.2% versus 9.6%; *p* < 0.001) and had a higher CURB-65 score than the no-AKI group. In addition, patients with AKI had higher levels of uric acid, serum Cystatin C, and white blood cell count, while they had lower levels of albumin, hemoglobin, and platelet count.

**Table 1.** Patients baseline in patients with and without acute kidney injury.

Variables	All (*n* = 2716)	Non-AKI (*n* = 1950)	AKI (*n* = 766)	*p* Value
Demographics				
Gender (male), *n* (%)	1653 (60.9)	1138 (58.4)	515 (67.2)	<0.001
Age (years)	71.6 ± 15.8	69.0 ± 16.2	78.2 ± 12.3	<0.001
Comorbid conditions, *n* (%)				
Hypertension	1380 (50.8)	903 (46.3)	477 (62.3)	<0.001
Diabetes mellitus	553 (20.4)	346 (17.7)	207 (27.0)	<0.001
Coronary artery disease	779 (28.7)	481 (24.7)	298 (38.9)	<0.001
Cardiac insufficiency	618 (22.8)	335 (17.2)	283 (36.9)	<0.001
Atrial fibrillation	317 (11.7)	182 (9.3)	135 (17.6)	<0.001
COPD	336 (12.4)	234 (12.0)	102 (13.3)	0.349
Chronic kidney disease	184 (6.8)	71 (3.6)	113 (14.8)	<0.001
Pulmonary hypertension	87 (3.2)	57 (2.9)	30 (3.9)	0.186
Tumor	238 (8.8)	159 (8.2)	79 (10.3)	0.073
Cerebrovascular diseases	883 (32.5)	535 (27.4)	348 (45.4)	<0.001
Complication				
Acute respiratory failure	487 (17.9)	187 (9.6)	300 (39.2)	<0.001
Severity scoring				
CURB-65 scores				<0.001
0	587 (21.6)	561 (28.8)	26 (3.4)	
1	1211 (44.6)	916 (47.0)	294 (38.6)	
2	741 (27.3)	416 (21.3)	323 (42.4)	
≥3	177 (6.5)	56 (2.9)	121 (15.8)	
Laboratory tests				
Baseline SCr (mmol/L)	64 (51–82)	62 (51–78)	69 (50–103)	<0.001
SCr (mmol/L)	74 (58–98)	68 (55–83)	104 (76–151)	<0.001
Albumin (g/L)	33.2 ± 5.3	34.2 ± 4.8	30.7 ± 5.4	<0.001
Uric acid (umol/L)	257 (186–360)	239 (178–314)	345 (228–477)	<0.001
Cystatin C (mg/L)	1.3 ± 0.7	1.1 ± 0.4	1.9 ± 0.9	<0.001
Hemoglobin (g/L)	118.4 ± 21.6	121.3 ± 19.5	111.0 ± 24.8	<0.001
Platelet count (10^9^/L)	198 (148–255)	188 (147–228)	155 (121–203)	<0.001
White blood cell count (10^9^/L)	7.4 (5.5–10.1)	6.9 (5.3–9.2)	8.8 (6.2–12.7)	<0.001

AKI: acute kidney injury; COPD: chronic obstructive pulmonary disease; CURB-65: confusion, urea nitrogen, respiratory rate, blood pressure, and age ≥65 years; SCr: serum creatinine.

### 3.3. Independent factors for AKI

Multivariate logistic regression analysis revealed that serum Cystatin C (odds ratio [OR] 4.27, 95% confidence interval [CI] 3.36–5.44; *p* < 0.001), acute respiratory failure (OR 3.96, 95% CI 2.29–3.83; *p* < 0.001), albumin (OR 0.91, 95% CI 0.89–0.94; *p* < 0.001), uric acid (OR 1.002, 95% CI 1.001–1.003; *p* < 0.001), platelet count (OR 0.997, 95% CI 0.996–0.998; *p* = 0.001), white blood cell count (OR 1.08, 95% CI 1.05–1.10; *p* < 0.001), and CURB-65 score were independent factors for AKI in patients with CAP (Table 2).

**Table 2.** Independent factors of AKI in patients with CAP.

Variable	OR	95% CI	*p* Value
Cystatin C (mg/L)	4.27	3.36–5.44	<0.001
Albumin (g/L)	0.91	0.89–0.94	<0.001
Uric acid (umol/L)	1.002	1.001–1.003	<0.001
Platelet count (10^9^/L)	0.997	0.996–0.998	0.001
White blood cell count (10^9^/L)	1.08	1.05–1.10	<0.001
CURB-65 scores			
0	Reference		
1	3.03	1.82–5.05	<0.001
2	4.10	2.38–7.08	<0.001
≥3	7.14	3.74–13.62	<0.001
Acute respiratory failure	2.96	2.29–3.83	<0.001

AKI: acute kidney injury; CAP: community-acquired pneumonia; OR: odds ratio; CI: confidence interval; CURB-65: confusion, urea nitrogen, respiratory rate, blood pressure, and age ≥ 65 years.

### 3.4. Prediction efficiency of serum Cystatin C for AKI in patients with CAP

**Figure 2.**
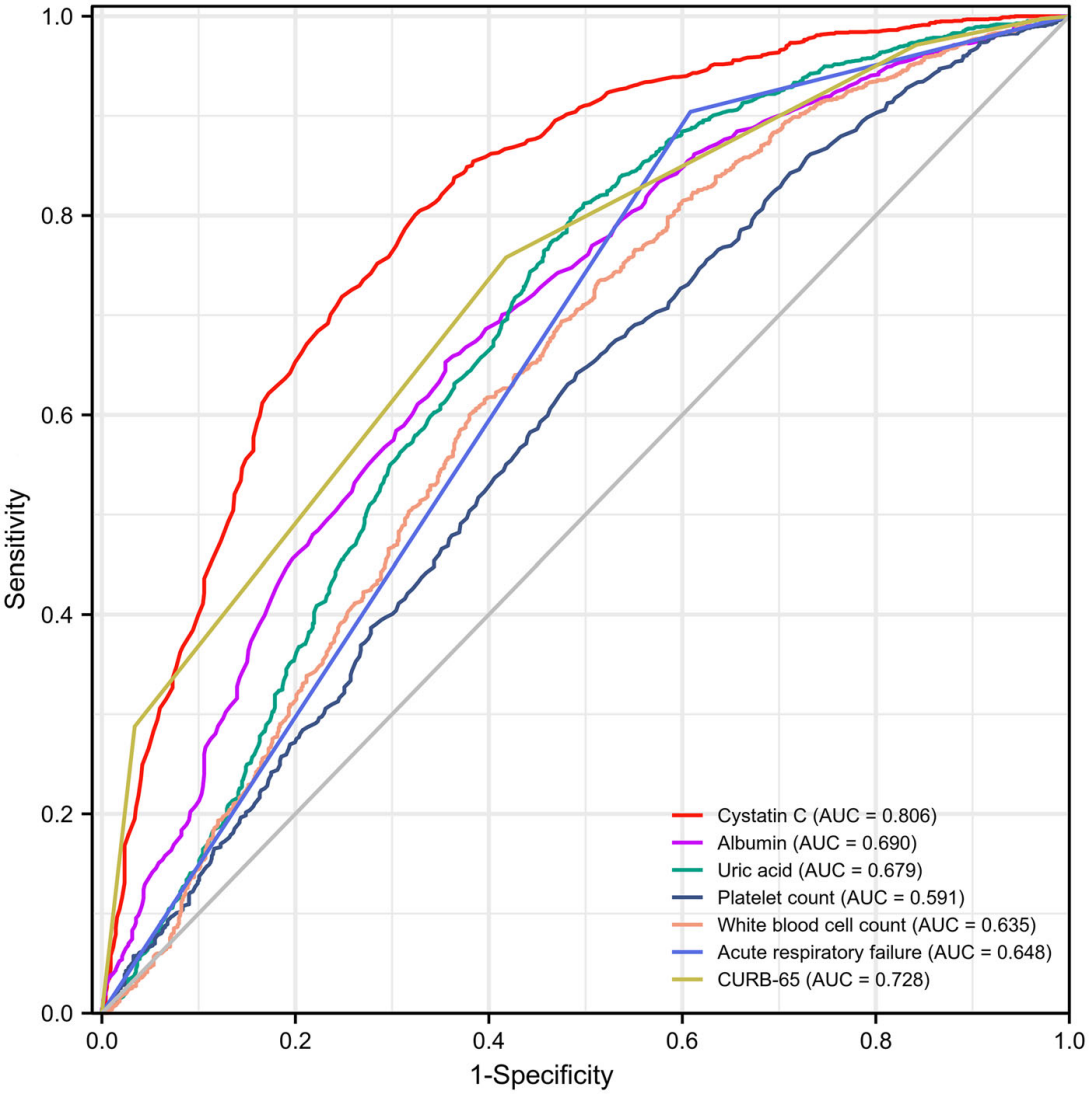
Comparisons of AUCs for all independent factors of AKI in patients with CAP. AUC: area under the receiver operating characteristic curve; AKI: acute kidney injury; CAP: community-acquired pneumonia.

We performed ROC analysis for all independent factors for AKI to determine their prediction performance, respectively. Figure 2 showed the comparisons of AUCs for all independent factors of AKI in patients with CAP. The maximum AUC was reported for serum Cystatin C within 24 h after admission. Table 3 presented the accuracy of serum Cystatin C for detecting AKI in patients with CAP. Serum Cystatin C had an AUC of 0.81 (95% CI: 0.79–0.83, *p* < 0.001) for predicting AKI, with an optimal cutoff value of 1.37 mg/L, computing 68% sensitivity, 80% specificity, 57% positive predictive value and 86% negative predictive value.

**Table 3.** The accuracy of serum Cystatin C for detecting AKI in patients with CAP.

AUC 95% CI	Cutoff point	Sensitivity	Specificity	Accuracy	PPV	NPV
0.81 (0.79–0.83)	1.37 mg/L	68%	80%	77%	57%	86%

AKI: acute kidney injury; CAP: community-acquired pneumonia; AUC: area under the receiver operating characteristic curve; CI: confidence interval; PPV: positive predictive value; NPV: negative predictive value.

### 3.5. Subgroup analysis

**Figure 3.**
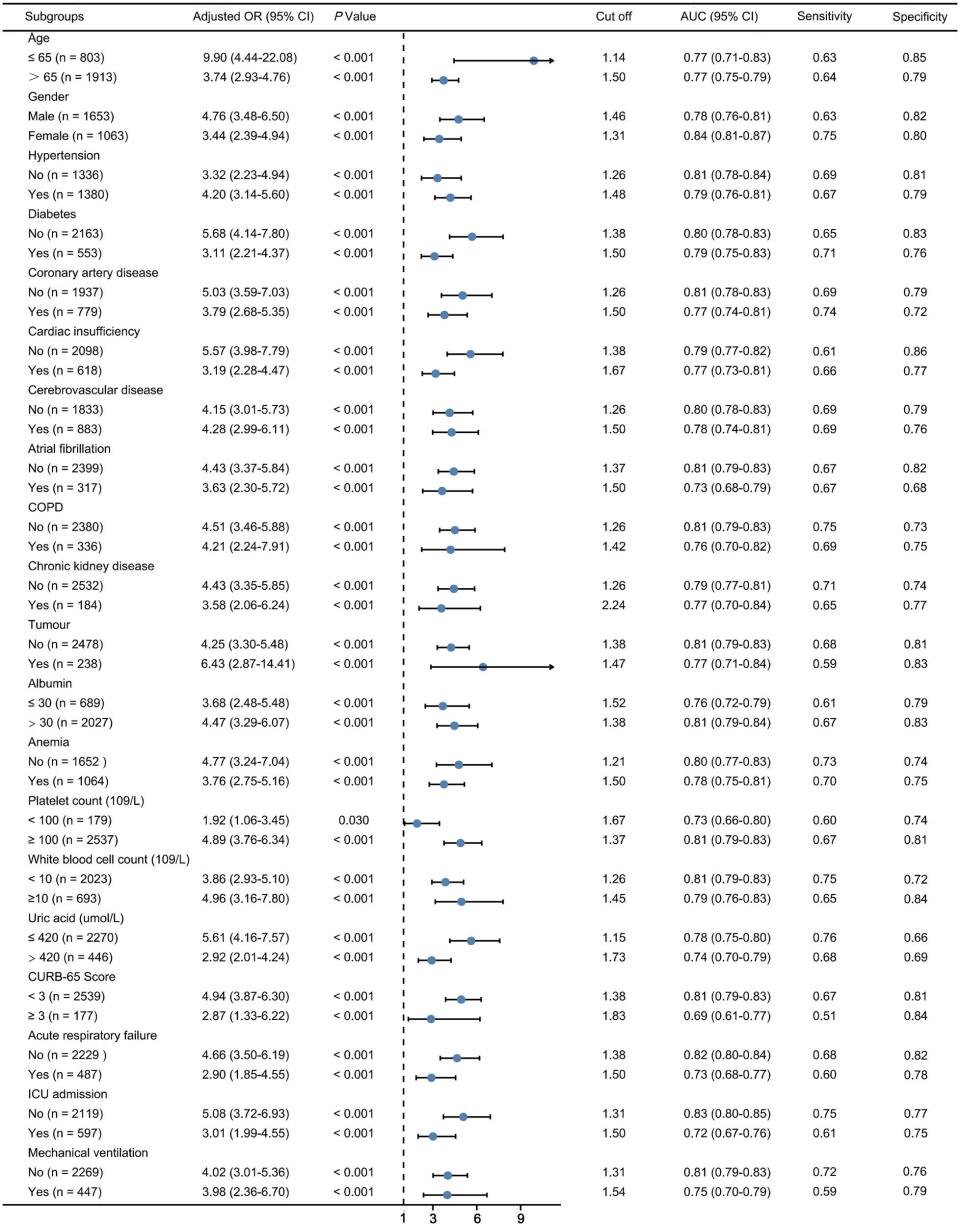
Prediction efficiency of serum Cystatin C for AKI in various subgroups of patients with CAP. AKI: acute kidney injury; CAP: community-acquired pneumonia.

In the stratification analyses, serum Cystatin C within 24 h after admission was still an independent predictor in all the various subgroups. Figure 3 showed the ORs, AUCs, cutoff values, sensitivity, and specificity of serum Cystatin C in all the different subgroups. Moreover, serum Cystatin C still had a good performance for predicting AKI in all the subgroups (AUCs: 0.69–0.84).

## 4. Discussion

Previously, serum Cystatin C had been demonstrated to be a biomarker for the early detection AKI in other clinical settings, such as patients with traumatic brain injury, neonates, patients with cardiac surgery, patients with liver, and so on [9,17–20]. To our best knowledge, this present study analyzed the largest number of Chinese patients with CAP to investigate serum Cystatin C within 24 h after admission for predicting AKI, and it indeed proved to be a good predictor of AKI in patients with CAP.

In this study, the incidence rate of AKI was 28%. Previous studies had demonstrated similar incidence rates of AKI in patients with CAP, ranging from 18 to 34% [2–4]. We found that serum Cystatin C was an important independent factor for predicting AKI in patients with CAP. Cystatin C is a 13 kDa proteinase inhibitor, and it is a member of the cystatin superfamily of cysteine protease inhibitors, which play an important role in intra-cellular catabolism of proteins and peptides [21]. It is synthesized and released into plasma by all nucleated cells at a constant rate [22]. Cystatin C can be more than 99% freely filtered through glomeruli and does not show significant protein binding. It is considered to be neither actively secreted into the tubular lumen nor reabsorbed into the plasma. After filtration, it is normally completely reabsorbed by proximal renal tubular epithelial cells, and catabolized by megalin receptor-induced endocytosis [23]. In addition, unlike SCr, the level of serum Cystatin C is not affected by age, sex, body muscle mass, and diet [9]. Therefore, serum Cystatin C is considered a good biomarker for the early detection of AKI. In our study, AUC of serum Cystatin C level to predict AKI was 0.81 (95% CI, 0.79–0.83), with an optimal cutoff value of 1.37 mg/L, computing 68% sensitivity, 80% specificity, 57% positive predictive value and 86% negative predictive value. Recently, Cigdem et al. conducted a single-centre, retrospective, observational cohort study with 348 hospitalized COVID-19 patients, and found that serum Cystatin C showed a good predictive power for AKI in patients with COVID-19. The AUC value of serum Cystatin C to predict COVID-19-related AKI was 0.96 (0.90 to 1.0), with the best cutoff value of 1.00 mg/L [24]. Albina et al. prospectively enrolled 341 patients presenting to the emergency department with CAP, and investigated the potential of plasma N-terminal prohormone B-type natriuretic peptide (NT-proBNP), mid-regional pro-atrial natriuretic peptide (MR-proANP) and B-type natriuretic peptide (BNP) levels within the first 48 h to predict early AKI in hospitalized patients with CAP. They found that the AUCs for the prediction of AKI of NT-proBNP, MR-proANP and BNP were 0.79, 0.79, and 0.74, respectively [25].

In this study, we also found that albumin, uric acid, platelet count, white blood cell count, CURB-65 score, and acute respiratory failure were independent factors for AKI in patients with CAP. CURB-65 [15] and the pneumonia severity index (PSI) [26] were two scoring systems that assessed the severity of CAP. Ahsan et al. reported that PSI was an important factor in predicting AKI in patients with CAP [2]. In our study, we found CURB-65 was also an independent risk factor to develop AKI in patients with CAP. Low albumin levels had been reported that it was a modifiable risk factor linked to increased risk of AKI in different clinical settings [27]. Recently, a study found that hypoalbuminemia was independently associated with the occurrence of AKI in COVID-19 patients with acute respiratory distress syndrome [28]. Albumin could improve renal perfusion and glomerular filtration by inhibiting apoptosis in renal tubular cells by carrying protective lysophosphatidic acid and scavenging reactive oxygen species [29]. Albumin could prolong potent renal vasodilation, which was induced by serum albumin reacting with the oxides of nitrogen to form S-nitroso-albumin [30]. In addition, several studies suggested that exogenous albumin administration was beneficial to protect the kidneys from AKI [31,32]. Hyperuricemia was associated with AKI in various statuses [33]. A recent report showed that serum uric acid was an independent predictor of AKI in patients with COVID-19 [34]. Platelet count and white blood cell count were two biomarkers for AKI, and a low level of platelet count and a high level of white blood cell count could increase the risk of AKI [35–38], which was consistent with our findings.

The present study had several strengths. First, to our best knowledge, this study may first report that serum Cystatin C within 24 h after admission could well predict AKI in patients with CAP. Second, stratified analyses made the use of data better. In subgroup analysis, serum Cystatin C within 24 h after admission still had a good prediction efficiency in different subgroups (AUCs: 0.69–0.84). Third, interestingly, serum Cystatin C had slightly better prediction performance for AKI in the subgroups of the non-elderly, female, and patients without the serious condition.

We acknowledge several limitations. First, it is a retrospective single-center study, and the conclusion will need to be confirmed by a multicenter prospective study with a larger patient cohort. Second, AKI is defined according to the KDIGO criteria, based on SCr and urine output [13]. However, the urine output data could not be obtained. Therefore, our analysis lacks the urine output standard for AKI. Third, Staphylococcus infections have increased the risk of AKI and are associated with mortality [39]. However, most of the patients in this study lack this data. Forth, we exclude the patients without sufficient SCr in the present study, and the potential selection bias might have also limited the generalizability of the study.

In summary, AKI is common in patients with CAP. The level of serum Cystatin C within 24 h after admission appears to be a good biomarker for predicting AKI in patients with CAP.

## Data Availability

The data underlying this article will be shared on reasonable request to the corresponding author.
